# Prevalence of metabolic dysfunction-associated steatotic liver disease (MASLD) and its association with arterial stiffness in adolescents: Results from the EVA4YOU study

**DOI:** 10.1371/journal.pone.0314585

**Published:** 2024-11-27

**Authors:** Johannes Nairz, Alex Messner, Sophia Zollner-Kiechl, Bernhard Winder, Christoph Hochmayr, Julian Granna, Alexander E. Egger, Andrea Griesmacher, Ralf Geiger, Michael Knoflach, Ursula Kiechl-Kohlendorfer, the Early Vascular Ageing in the YOUth (EVA4YOU) Study Group

**Affiliations:** 1 VASCage Research Centre on Vascular Ageing and Stroke, Innsbruck, Tyrol, Austria; 2 Department of Pediatrics II, Medical University of Innsbruck, Innsbruck, Tyrol, Austria; 3 Department of Pediatrics III, Medical University of Innsbruck, Innsbruck, Tyrol, Austria; 4 Department of Neurology, Hochzirl Hospital, Zirl, Tyrol, Austria; 5 Department of Vascular Surgery, Feldkirch Hospital, Feldkirch, Vorarlberg, Austria; 6 Central Institute of Medical and Chemical Laboratory Diagnostics (ZIMCL), University Hospital of Innsbruck, Innsbruck, Tyrol, Austria; 7 Department of Neurology, Medical University of Innsbruck, Innsbruck, Tyrol, Austria; Universita degli Studi della Campania Luigi Vanvitelli Scuola di Medicina e Chirurgia, ITALY

## Abstract

**Aim:**

To determine the prevalence of metabolic dysfunction-associated steatotic liver disease (MASLD) among Western Austrian adolescents and its association with arterial stiffness as a marker of early vascular ageing.

**Methods:**

In the cross-sectional Early Vascular Ageing in the YOUth study, liver fat content was assessed by controlled attenuation parameter (CAP) using signals acquired by FibroScan (Echosense, Paris, France) in 14- to 19-year-old Austrian adolescents. Arterial stiffness was determined by carotid-femoral pulse wave velocity (cfPWV) and cardiovascular risk factors by a face-to-face interview, physical examination, and fasting blood analyses. Linear regression models and one-way analysis of variance were performed to analyze the association between liver fat content, MASLD and cfPWV.

**Results:**

A total of 1292 study participants (65.2% female) aged 17.2 ± 1.3 years were included. MASLD was detected in 62 (4.8%) adolescents. CAP value showed a significant association with cfPWV in the unadjusted model (p < 0.001) but lost its significant influence in the multivariable model after adjusting for sex, age and cardiovascular risk criteria (increased BMI or waist circumference, impaired glucose metabolism, elevated blood pressure, elevated plasma triglycerides, and decreased HDL cholesterol; p = 0.540). In the analysis of variance, a significant increase in cfPWV was observed in adolescents with any of the five cardiovascular risk criteria for MASLD (p < 0.001), but not with the additional presence of steatotic liver disease (p = 0.291).

**Conclusion:**

In our adolescent cohort, liver fat content and MASLD were not found to be independent predictors for early vascular ageing. Nevertheless, the determination of liver fat content can be a useful tool to identify adolescents at high risk for cardiovascular disease and metabolic syndrome.

## Introduction

There is increasing evidence that arteriosclerosis, leading to cardiovascular disease (CVD) later in life, starts early in childhood and is already associated with the presence of cardiovascular risk factors, such as dyslipidemia, obesity, diabetes, and hypertension in this age group [1, 2]. Non-alcoholic fatty liver disease (NAFLD) is discussed as a further cardiovascular risk factor in adolescents, similarly as it appears to be in adults [3]. Even though NAFLD is strongly associated with atherosclerosis and CVD [4], it is unclear whether this effect is attributable to the strong correlation of steatotic liver disease (SLD) and cardiometabolic factors including obesity, impaired glucose metabolism, and dyslipidemia [5, 6]. In 2023, three large multinational liver associations gave additional weight to the strong association of SLD with cardiometabolic factors by replacing the terminology NAFLD with ‘metabolic dysfunction-associated steatotic liver disease’ (MASLD) and making the presence of at least one out of five cardiometabolic criteria mandatory for the diagnosis of MASLD [7] (for detailed diagnostic criteria see section Liver fat quantification and assessment of MASLD). So far, no data on the prevalence of MASLD in European adolescents has been published.

Arterial stiffness is a marker of atherosclerosis and early vascular ageing [8] and can be determined by carotid-femoral pulse wave velocity (cfPWV), which increases in stiffer arteries [9]. In a cohort of 17-year-olds, Huang et al. found an influence of steatotic liver on cfPWV only in those who belonged to a “high-risk” metabolic cluster, whereas there was no association in those who had no other cardiometabolic risk factor, leading them to conclude that steatotic liver is not an independent risk factor for arterial stiffness *per se* [10]. So far, there has not been any other published study investigating the relationship between steatotic liver, MASLD and cfPWV in adolescents.

In this study, we aimed to describe the prevalence of MASLD in a cohort of 14- to 19-year-old Austrian adolescents within the Early Vascular Ageing in the YOUth (EVA4YOU) study and investigate the influence of the degree of fatty liver and MASLD on arterial stiffness.

## Methods

### Study population

The EVA4YOU study is a single-center cross-sectional study, conducted between 01/02/2021 and 31/03/2023 at 57 schools and companies all over Tyrol, a state in western Austria with approximately 760 000 inhabitants, primarily of Caucasian descent [6, 11, 12]. We assessed the health status of Tyrolean adolescents aged 14 to 19 years focusing on cardiovascular risk factors and manifestations of early vascular ageing, including liver fat quantification and cfPWV.

All participants signed a written informed consent form, and if the participants were under the age of 18, consent was additionally given by their legal representatives. There were no exclusion criteria apart from a lack of written informed consent or absence on the day of the examination. The study was approved by the ethics committee of the Medical University of Innsbruck (approval number: 1053/2020) and was conducted in accordance with the Declaration of Helsinki.

### Liver fat quantification and assessment of MASLD

Liver fat content was assessed by the controlled attenuation parameter (CAP) using signals acquired by FibroScan (Echosense, Paris, France), which is a standardized non-invasive measurement of hepatic steatosis [13]. The measurement was performed with the participant in a supine position with the right arm under the head and the right leg crossed over the left, causing a bend to the left side. The FibroScan M probe was positioned vertically on the patient’s skin surface in an intercostal space along the medial axillary line, using the xiphoid process as a reference point for determining an appropriate intercostal space over the center of the right hepatic lobe. Results were only included in the final analysis if the patient was fasting for at least 3 hours prior to the examination and more than 10 complete measurements were available. The device estimates hepatic steatosis in decibels/meter (dB/m) within a range of 100–400 dB/m.

To determine manifest SLD, we used the 90^th^ percentile threshold for CAP values from a reference dataset [14]. NAFLD was defined as SLD in the absence of excessive alcohol consumption (< 140 g per week for women and < 210 g per week for men) and any other known specific cause of hepatic steatosis [5]. For making the diagnosis of MASLD, we used the new definition criteria of the multisociety Delphi consensus statement [7]. In brief, this includes all participants with manifest SLD without underlying causal liver pathology (including excessive alcohol consumption, as stated above) and at least one out of five cardiometabolic criteria, which are:

- Body mass index (BMI) ≥ 85^th^ percentile or waist circumference > 95^th^ percentile.
- Fasting serum glucose ≥ 5.6 mmol/L or serum glucose ≥ 11.1 mmol/L or 2-hour post-load glucose levels ≥ 7.8 mmol or glycated hemoglobin (HbA1c) ≥ 5.7% or already diagnosed/treated type 2 diabetes.
- Blood pressure ≥ 130/85 mmHg or specific antihypertensive drug treatment.
- Plasma triglycerides ≥ 1.70 mmol/L or lipid lowering treatment.
- Plasma high-density lipoprotein (HDL) cholesterol ≤ 1.0 mmol/L or lipid lowering treatment.

To determine a correlation with arterial stiffness, CAP value was used as a continuous variable for the linear regression model and as a categorical variable for the diagnosis of MASLD for the one-way analysis of variance (see section Statistical analysis).

### Carotid-femoral pulse wave velocity (cfPWV)

Measuring cfPWV is the gold standard for determining aortic stiffness and is considered an independent predictor of CVD in the general population [15]. We used the Vicorder device (80 Beats Medical GmbH, Berlin, Germany) according to the manufacturer’s instructions. In brief, subjects were in supine position, head and shoulders elevated by 30°, one cuff placed over the right carotid artery and another over the right femoral artery to simultaneously record the carotid and femoral pulse waves by plethysmographic oscillometry. An algorithm selected the rapidly rising lower parts of all systolic sections of the proximal and distal waves from ten consecutive artefact-free pulse waves to determine the transit time of the pressure wave. Travel distance was measured to the nearest 0.1 cm over the surface of the body between the suprasternal notch and the center of the femoral cuff using a non-stretchable tape. Finally, cfPWV was calculated as the travel distance divided by the carotid-femoral transit time in m/s. The mean value of two cfPWV measurements was used for our analysis.

### Laboratory methods

Blood samples were taken in the morning after an overnight fasting period, cooled and immediately delivered to the ISO 15189 accredited Central Institute of Medical and Chemical Laboratory Diagnostics (University Hospital Innsbruck, Austria). HDL cholesterol, triglycerides, and glucose were assessed by enzymatic colorimetric assays (Cobas 8000, Roche Diagnostics, Rotkreuz, Switzerland) and HbA1c was measured by high-pressure liquid chromatography (Tosoh G8, Tosoh Bioscience, Tessenderlo, Belgium). Fasting glucose was defined as serum glucose levels after a fasting period of at least 8 hours.

### Alcohol intake

Alcohol intake was assessed in a face-to-face interview carried out by physicians. It was calculated in grams per week by the product of alcohol content, volume and intake frequency.

### Anthropometry

Body height and weight were measured using calibrated scales, and BMI was calculated as body weight in kilograms divided by the square of height in meters. Waist circumference was measured with a non-stretchable tape to the nearest 0.1 cm halfway between the iliac crest and the lower chest after a slight expiration. Systolic and diastolic blood pressure was calculated as the mean of three measurements on the left and right arm taken after a 5-minute seated rest using an automatic oscillometer device (OMRON M4-I, Omron Healthcare Co., Lake Forest, Illinois, USA). All anthropometric measurements were converted into age and sex specific z-scores according to German reference datasets [16–18].

### Statistical analysis

Characteristics of the study cohort are shown as count (percentage) for categorial variables and mean ± SD for continuous variables. Between-group differences were determined using the Student t test, Fisher exact test, and χ2 test, depending on the type and distribution of the variable analyzed.

Linear regression models were performed to determine the association between CAP value and cfPWV. Model 1 was unadjusted. Model 2 was adjusted for sex and age. Model 3 was adjusted for sex, age, and the cardiovascular risk criteria for MASLD defined in the Delphi consensus statement [7] (increased BMI or waist circumference, impaired glucose metabolism, elevated blood pressure, elevated plasma triglycerides, and decreased HDL cholesterol levels). For all models, the assumptions (independence and normality of the errors, homoscedasticity, and linearity of the relationship between dependent and independent variables) were tested and satisfied. Collinearity was validated by variance inflation factor, which was < 2 for all included variables.

Furthermore, we aimed to determine the impact of MASLD on cfPWV independently of the five cardiometabolic risk criteria for MASLD. For this analysis, three groups were formed: Participants who neither fulfilled the cardiometabolic criteria for MASLD nor had SLD formed group 1. Group 2 included those who had at least one out of the five determined cardiometabolic criteria but no SLD, and finally group 3 included those who fulfilled both the cardiometabolic criteria for MASLD and had SLD. All subjects who had an underlying causal liver pathology for SLD (including those with excessive alcohol consumption) were excluded for this analysis, thus group 3 met all definition criteria for MASLD. The differences in cfPWV between the mentioned groups were determined using one-way analysis of variance (ANOVA) and post-hoc Bonferroni statistical tests.

All analyses were conducted using SPSS version 29.0 (SPSS Inc., Chicago Illinois, USA). Hypotheses tests were two-sided and P values were considered statistically significant at p < 0.05.

## Results

**Fig 1:**
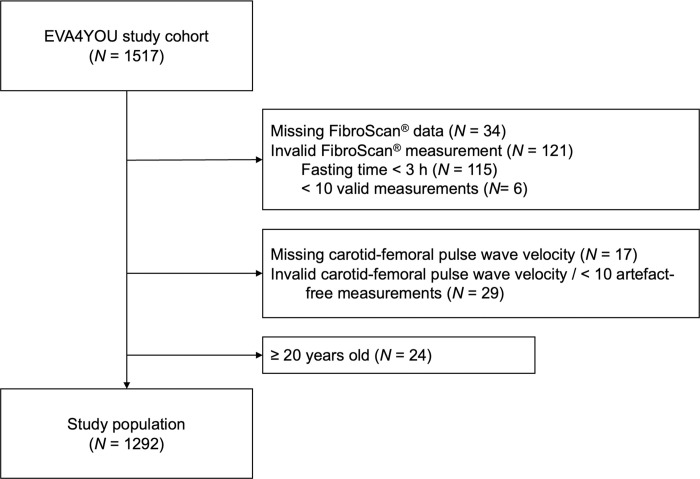
Study flow chart.

1517 adolescents were included in the EVA4YOU study. 155 participants were excluded due to missing or invalid FibroScan measurements, 46 due to missing or invalid cfPWV measurements and 24 adolescents were excluded due to an age of 20 years or older. Therefore, our cohort consisted of 1292 participants (see flow chart in Fig 1).

Detailed characteristics of the study cohort are shown in Table 1. Adolescents were on average 17.2 ± 1.3 years old and 65.2% were female (no participant reported a gender other than male or female). The mean cfPWV was 6.0 ± 0.7 m/s. Using the 90^th^ percentile threshold from a reference dataset [14], 69 (5.3%) participants in our cohort had manifest SLD and 67 (5.2%) met the diagnostic criteria for NAFLD. According to the new Delphi consensus statement [7], 62 (4.8%) study participants fulfilled the diagnostic criteria for MASLD (3.8% in females, 6.7% in males). In addition, 1 participant (0.1%) met the diagnostic criteria for metabolic dysfunction and alcohol associated steatotic liver disease (MetALD), 1 participant (0.1%) met those for alcohol-associated/related liver disease (ALD) and 5 participants had cryptogenic SLD. As expected, there are some significant differences in the characteristics between males and females (Table 1) and between those with and without MASLD (S1 Table).

**Table 1:** Characteristics of the study population.

	All	Males	Females	P value
	*N* = 1292 (100%)	*N* = 450 (34.8%)	*N* = 842 (65.2%)	
**Demographics**				
Age, y	17.2 ± 1.3	17.3 ± 1.3	17.2 ± 1.3	0.431^a^
**Pulse wave velocity**				
cfPWV, m/s	6.0 ± 0.7	6.4 ± 0.7	5.8 ± 0.6	**<0.001** ^**a**^
**Alcohol intake**				
Alcohol intake ≥ 140 g/week (females) / ≥ 210 g/week (males)	51 (3.9%)	30 (6.7%)	21 (2.5%)	**<0.001** ^**b**^
> 350 g/week (females) / > 420 g/week (males)	7 (0.5%)	7 (1.6%)	0 (0%)	**<0.001** ^**c**^
**Cardiometabolic risk factors**				
Overweight/obese^d^	209 (16.2%)	83 (18.4%)	126 (15.0%)	0.106^b^
BMI, kg/m^2^	22.2 ± 3.6	22.5 ± 3.5	22.0 ± 3.6	**0.036** ^**a**^
Impaired glucose metabolism^e^	58 (4.5%)	20 (4.4%)	38 (4.5%)	0.964^b^
Arterial hypertension^f^	501 (38.8%)	246 (54.7%)	255 (30.3%)	**<0.001** ^**b**^
Elevated plasma triglycerides^g^	83 (6.4%)	22 (4.9%)	61 (7.2%)	0.100^b^
Decreased HDL cholesterol^h^	68 (5.3%)	40 (8.9%)	28 (3.3%)	**<0.001** ^**b**^
**Steatotic liver disease**				
CAP, dB/m	187.3 ± 40.1	199.0 ± 37.7	181.1 ± 40.0	**<0.001** ^**a**^
CAP ≥ 90^th^ percentile^i^	69 (5.3%)	33 (7.3%)	36 (4.3%)	**0.020** ^**b**^
MASLD^j^	62 (4.8%)	30 (6.7%)	32 (3.8%)	**0.022** ^**b**^

Values are given as mean ± SD or count (%). Missing data were < 2% for all assessed parameters.

cfPWV, carotid-femoral pulse wave velocity; BMI, body mass index; HDL, high-density lipoprotein; CAP, controlled attenuation parameter; MASLD, metabolic dysfunction-associated steatotic liver disease; and SLD, steatotic liver disease.

^a^ Student t test.

^b^ χ^2^ test.

^c^ Fisher exact test.

^d^ BMI ≥ 85th percentile or waist circumference > 95th percentile.

^e^ Fasting serum glucose ≥ 5.6 mmol/L or serum glucose ≥ 11.1 mmol/L or 2-hour post-load serum glucose ≥ 7.8 mmol or HbA1c ≥ 5.7% or already diagnosed/treated type 2 diabetes.

^f^ Blood pressure ≥ 130/85 mmHg or antihypertensive drug treatment.

^g^ Plasma triglycerides ≥ 1.70 mmol/L or lipid lowering treatment.

^h^ HDL cholesterol ≤ 1.0 mmol/L or lipid lowering treatment.

^i^ Threshold for manifest SLD; calculated using a reference data set [14].

^j^ Defined as SLD without underlying causal liver pathology (including excessive alcohol consumption) and with at least one out of five cardiometabolic risk factors (see also in section Liver fat quantification and assessment of MASLD).

The linear regression models to determine the association between CAP value and cfPWV are shown in Table 2. In this analysis, CAP value showed a significant positive association with cfPWV in the unadjusted model (p < 0.001) but lost its significant influence after adjustment for sex and age (p = 0.309). Additional adjustment for cardiovascular risk factors improved the goodness of fit of the model (adjusted ΔR^2^ = 0.028), while the influence of CAP value on cfPWV remained non-significant (p = 0.540).

**Table 2:** Association between CAP value and carotid-femoral pulse wave velocity.

	Model 1		Model 2		Model 3	
	unadjusted		adjusted for sex and age		adjusted for sex, age and cardiometabolic risk criteria	
	(adjusted R^2^ = 0.016)		(adjusted R^2^ = 0.228)		(adjusted R^2^ = 0.256)	
	Regression coefficient (95% CI)	P value	Regression coefficient (95% CI)	P value	Regression coefficient (95% CI)	P value
CAP, dB/m	0.002 (0.001, 0.003)	**<0.001**	0.000 (0.000, 0.001)	0.309	0.000 (-0.001, 0.001)	0.540

Linear regression models. Model 1 is unadjusted. Model 2 with adjustment for sex and age. Model 3 with adjustment for sex, age, and the cardiometabolic risk criteria for MASLD (BMI ≥ 85^th^ percentile or waist circumference > 95^th^ percentile; Fasting serum glucose ≥ 5.6 mmol/L or serum glucose ≥ 11.1 mmol/L or 2-hour post-load glucose levels ≥ 7.8 mmol or glycated hemoglobin (HbA1c) ≥ 5.7% or already diagnosed/treated type 2 diabetes; Blood pressure ≥ 130/85 mmHg or specific antihypertensive drug treatment; Plasma triglycerides ≥ 1.70 mmol/L or lipid lowering treatment; Plasma high-density lipoprotein cholesterol ≤ 1.0 mmol/L or lipid lowering treatment).

CAP, controlled attenuation parameter; and MASLD, metabolic dysfunction-associated steatotic liver disease.

**Fig 2:**
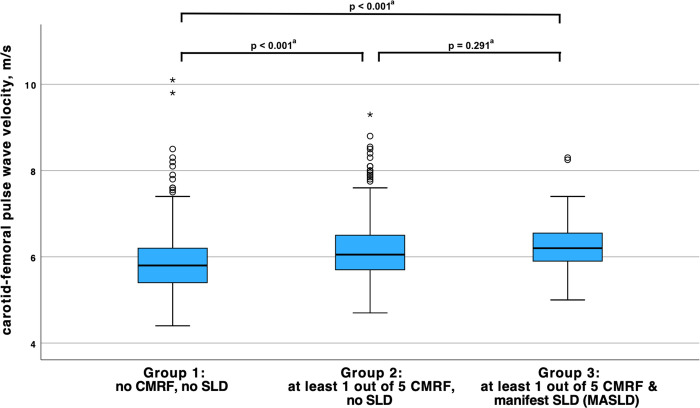
Boxplot mean cfPWV comparisons. Subjects with an underlying causal liver pathology for SLD (including excessive alcohol consumption) were excluded from this analysis (N = 51), thus participants from group 3 met all definition criteria for MASLD. Moderate outliers are marked with circles (o) and extreme outliers are marked with asterisks (*). CMRF, cardiometabolic risk factor; SLD, steatotic liver disease; and MASLD, metabolic dysfunction-associated steatotic liver disease. a Pairwise comparison of the means using post-hoc Bonferroni statistical tests (one-way ANOVA).

Subsequently, one-way ANOVA and Bonferroni-adjusted post-hoc analysis was performed to determine the difference in cfPWV values between the three formed groups (see section Statistical analysis). Mean cfPWV in group 1 (participants without cardiometabolic risk factor and no SLD) was 5.9 ± 0.7 m/s, in group 2 (participants with cardiometabolic risk factors but no SLD) 6.1 ± 0.7 m/s and in group 3 (participants with cardiometabolic risk factors and SLD, i.e. meeting all MASLD definition criteria) 6.3 ± 0.7 m/s. There was an overall significant difference in cfPWV between these three groups (F(2,1228) = 30.787, R^2^ = 0.046, p < 0.001), but finally, in the post hoc analysis, between group 2 and group 3 no significant difference in cfPWV was found (p = 0.291; see Fig 2). Therefore, the additional presence of SLD in individuals who met one of the five cardiometabolic criteria for MASLD did not result in an additional significant increase in cfPWV.

## Discussion

NAFLD and CVD share many metabolic features and risk factors [19]. This overlap prompted an international panel of experts to redefine NAFLD as metabolic dysfunction-associated steatotic liver disease (MASLD) [7]. An excellent concordance rate between NAFLD and MASLD definitions has been demonstrated, which means that about 99% of people with NAFLD also fulfill the new MASLD definition criteria [20]. In our adolescent cohort, we found a prevalence of MASLD equal to 4.8%, which corresponds to a concordance rate of 92.5% with NAFLD prevalence in our cohort (5.2%). In comparison, in the US, a recent study showed a higher prevalence of MASLD of 16.8% among 12- to a 17-year-old and 25.5% among 18- to 25-year-old adolescents [21], while in another adolescent US cohort a prevalence of 10.1% was reported [22]. These differences in prevalence could be due to genetic, epigenetic, or environmental factors including dietary habits and physical activity [23].

CVD is the leading cause of death among people with NAFLD [24]. In adults, it is widely recognized that the extent of liver fat increases CVD-related morbidity or mortality independent of traditional cardiovascular risk factors, whereas this association is still controversial in the pediatric population [3]. PWV is a marker for arterial stiffening, especially of the large conduit arteries, which is a major manifestation of vascular ageing [25]. Furthermore, longitudinal studies have shown that increased arterial stiffness in adolescence is associated with elevated insulin resistance, obesity, high blood pressure and metabolic syndrome in young adulthood [26–28]. In our study, liver fat content was only associated with cfPWV in the unadjusted model and lost its significant influence after adjusting for sex and age, known important predictors of cfPWV. Sex hormones appear to be the underlying cause when it comes to sex differences in arterial stiffness, as lower free testosterone levels in women and higher estradiol levels in men were associated with greater aortic distensibility in a previous study [29]. It is also known that this difference decreases with age and that at older ages women have higher aortic stiffness than men [30]. Again, a key role of estrogen in the age-related increase in aortic stiffness in women has been suggested [31]. In addition, arterial stiffness increases with age, because repeated pulsations lead to fatigue and rupture of the elastin lamellae of the central arteries [32].

Further adjustment for other cardiovascular risk factors improved the goodness of fit of our model, while CAP value remained non-significant. This finding is supported by the fact that participants with at least one cardiometabolic risk factor with SLD (= MASLD) or without SLD did not differ in cfPWV (Fig 2). We therefore conclude that liver fat content is not an independent risk factor for early vascular ageing in adolescence, but this is likely mediated by the strong association of cardiometabolic risk factors with both–SLD as well as cfPWV. In our previous work, we were able to demonstrate that SLD is strongly associated with ‘traditional’ cardiometabolic risk factors such as triglyceride levels, BMI and insulin resistance and represents the liver manifestation of metabolic syndrome [6].

The strengths of this study include the large sample size, the uniform measurement of cfPWV and CAP by experienced and trained personnel, and the broad data set including all cardiovascular risk criteria for MASLD. We enrolled adolescents from all regions of Tyrol and from all types of schools and companies, so our cohort can be considered representative of Tyrolean adolescents. To the best of our knowledge, this is the first study to determine the prevalence of MASLD according to the new definition criteria [7] in a European adolescent cohort and to analyze its association with cfPWV.

There are some limitations as well: We did not assess data regarding the pubertal stage. However, it can be assumed that almost all participants were in Tanner stage IV or V [33]. Furthermore, previous studies in 11 and 13-year-olds have shown that there is no influence of Tanner stage on atherosclerotic changes [34, 35]. The prevalence of elevated blood pressure was high in our cohort (38.8%), since we used the cut-off values defined for the diagnosis of MASLD (blood pressure ≥ 130/85 mmHg for ≥ 13-year-old subjects) [7]. Using the cut-off values according the 2023 European Society of Hypertension guidelines (diastolic and/or systolic blood pressure ≥ 95^th^ percentile for < 16-year-old subjects and systolic blood pressure ≥ 140 mmHg and/or diastolic blood pressure ≥ 90 mmHg for ≥ 16-year-old subjects) [36], 19.1% of our cohort would have arterial hypertension. To determine SLD, we used the CAP value measured with FibroScan, a non-invasive method with a sensitivity of 87% and a specificity of 91% for the detection of hepatic steatosis [37], while the gold standard would be liver histology [38]. However, as this study was conducted on a healthy adolescent population, invasive diagnostics cannot be performed for ethical reasons. In addition, as this is a cross-sectional study and we therefore measured arterial stiffness and SLD at the same time, we can only describe correlations but no conclusions about cause and effect can be drawn.

## Conclusion

In summary, our study of 14- to 19-year-old Western Austrian adolescents showed a MASLD prevalence of 4.8% (3.8% in females, 6.7% in males). In the unadjusted model, the extent of hepatic steatosis correlated significantly with arterial stiffness, a well-known marker for future cardiovascular risk and mortality in adults [15]. However, after adjustment for sex, age and the cardiometabolic risk criteria for MASLD, it lost its significance, leading us to conclude that hepatic steatosis is not an independent risk factor for early vascular ageing in our adolescent cohort.

Nevertheless, the determination of liver fat content in adolescents can be a useful tool, as even non-pharmacological approaches at this age can reduce and reverse the risk of severe hepatic steatosis and cirrhosis in young adulthood [39] and additionally it can be used to identify adolescents at high risk of cardiovascular disease and metabolic syndrome.

## Supporting information

S1 Checklist STROBE statement—checklist of items that should be included in reports of cross-sectional studies. (DOC)

S1 Table Characteristics of the study population (based on the presence or absence of MASLD). (DOCX)
