# Supplementary material for: Prevalence of metabolic dysfunction-associated steatotic liver disease (MASLD) and its association with arterial stiffness in adolescents: Results from the EVA4YOU study
Source: PLoS One. 2024 Nov 27;19(11):e0314585. doi: 10.1371/journal.pone.0314585 (PMC11602044; doi:10.1371/journal.pone.0314585)
Supplement: S1 Table — (DOCX) [file pone.0314585.s002.docx]

S1 Table. Characteristics of the study population (based on the presence or absence of MASLD)

|  | All^a^  *N* = 1285 (100%) | MASLD  *N* = 62 (4.8%) | no SLD  *N* = 1223 (95.2%) | P value |
| --- | --- | --- | --- | --- |
| **Demographics** |  |  |  |  |
| Age, y | 17.2 ± 1.3 | 17.9 ± 1.2 | 17.2 ± 1.3 | **<0.001^b^** |
| Sex, male | 447 (34.8%) | 30 (48.4%) | 417 (34.1%) | **0.021^c^** |
| **Pulse wave velocity** |  |  |  |  |
| cfPWV, m/s | 6.0 ± 0.7 | 6.3 ± 0.7 | 6.0 ± 0.7 | **<0.003^b^** |
| **Alcohol intake** |  |  |  |  |
| Alcohol intake ≥ 140 g/week (females) / ≥ 210 g/week (males) | 49 (3.8%) | 0 (0.0%) | 49 (4.0%) | 0.167^d^ |
| > 350 g/week (females) / > 420 g/week (males) | 6 (0.5%) | 0 (0.0%) | 6 (0.5%) | 0.999^d^ |
| **Cardiometabolic risk factors** |  |  |  |  |
| Overweight/obese^e^ | 208 (16.2%) | 52 (83.9%) | 156 (12.8%) | **<0.001^c^** |
| BMI, kg/m^2^ | 22.2 ± 3.6 | 28.9 ± 3.8 | 21.9 ± 3.2 | **<0.001^b^** |
| Impaired glucose metabolism^f^ | 58 (4.5%) | 6 (9.8%) | 52 (4.3%) | 0.053^d^ |
| Arterial hypertension^g^ | 499 (38.8%) | 37 (59.7%) | 462 (37.8%) | **<0.001^c^** |
| Elevated plasma triglycerides^h^ | 82 (6.4%) | 10 (16.4%) | 72 (5.9%) | 0.004^d^ |
| Decreased HDL cholesterol^i^ | 67 (5.2%) | 12 (19.7%) | 55 (4.5%) | **<0.001^d^** |

Values are given as mean ± SD or count (%). Missing data were < 2% for all assessed parameters.

MASLD, metabolic dysfunction-associated steatotic liver disease, SLD, steatotic liver disease; cfPWV, carotid-femoral pulse wave velocity; BMI, body mass index; HDL, high-density lipoprotein; MetALD, metabolic dysfunction and alcohol associated steatotic liver disease; and ALD, alcohol-associated/related liver disease.
^a^ Individuals with MetALD, ALD and cryptogenic SLD (*N* = 7) were excluded from this analysis.
^b^ Student t test.
^c^ χ^2^ test.
^d^ Fisher exact test.

^e^ BMI ≥ 85th percentile or waist circumference > 95th percentile.

^f^ Fasting serum glucose ≥ 5.6 mmol/L or serum glucose ≥ 11.1 mmol/L or 2-hour post-load serum glucose ≥ 7.8 mmol or HbA1c ≥ 5.7% or already diagnosed/treated type 2 diabetes.
^g^ Blood pressure ≥ 130/85 mmHg or antihypertensive drug treatment.
^h^ Plasma triglycerides ≥ 1.70 mmol/L or lipid lowering treatment.
^i^ HDL cholesterol ≤ 1.0 mmol/L or lipid lowering treatment.
